# Exercise and Carotid Properties in the Young–The KiGGS-2 Study

**DOI:** 10.3389/fcvm.2021.767025

**Published:** 2022-01-05

**Authors:** Karsten Königstein, Julia Charlotte Büschges, Giselle Sarganas, Susanne Krug, Hannelore Neuhauser, Arno Schmidt-Trucksäss

**Affiliations:** ^1^Department of Epidemiology and Health Monitoring, Robert Koch Institute, Berlin, Germany; ^2^Division Sports and Exercise Medicine, Department of Sport, Exercise and Health, University of Basel, Basel, Switzerland; ^3^DZHK (German Centre for Cardiovascular Research), Partner Site Berlin, Berlin, Germany

**Keywords:** intima-media thickness, arterial stiffness, atherosclerosis, adolescents, exercise, carotid stiffness

## Abstract

**Background:** Carotid intima-media thickness (cIMT) and stiffness (cS) are predictive markers of early vascular aging and atherosclerotic risk. This study assessed, whether exercise has protective effects on carotid structure and function or on vascular risk in the young.

**Methods:** Volume and change of exercise (recreational and organized sports participation) of German adolescents and young adults was assessed within the prospective population-study KiGGS at KiGGS-Wave-1 (2009–2012) and KiGGS-Wave-2 (2014–2017) using standardized self-reporting questionnaires. CIMT and cS were measured by real-time B-mode ultrasound sequences with semi-automated edge-detection and automatic electrocardiogram-gated quality control in 2,893 participants (14–28 years, 49.6% female). A cumulative index for atherosclerotic risk (CV-R) included z-scores of mean arterial pressure, triglycerides, total/HDL-cholesterol-ratio, body mass index, and HbA1c.

**Results:** At KiGGS-Wave-2 cross-sectional CV-R but not cS and cIMT was lower in all exercise-groups compared to “no exercise” (B = −0.73, 95%-CI = −1.26 to 0.19, *p* = 0.008). Longitudinal volume of exercise was negatively associated with CV-R (B = −0.37, 95%-CI = −0.74 to 0.00, *p* = 0.048) but not with cS and cIMT. Cross-sectional relative risk of elevated CV-R but not cS and cIMT was lower in all exercise-groups compared to “no exercise” (RR = 0.80, 95%-CI = 0.66 to 0.98, *p* = 0.033). High exercise volumes were associated with lower relative risk of elevated CV-R (RR = 0.80, 95%-CI = 0.65–0.97, *p* = 0.021) and cS in tendency but not with cIMT.

**Conclusions:** Increased levels of exercise are associated with a better cardiovascular risk profile in young individuals, but not with cS and cIMT. Our study confirms previous recommendations on exercise in this age group without demonstrating a clear benefit on surrogate markers of vascular health.

## Introduction

Vascular aging is characterized by endothelial dysfunction, increased arterial stiffness, and structural remodeling of the vascular walls (1). It is a lifelong process, leading to elevated risk of atherosclerotic disease at higher age (2, 3). This process is mainly driven by chronic low-grade inflammatory activity (4, 5) and accelerated by atherosclerotic risk factors (6), i.e., obesity (7), physical inactivity (8), western type diet (9), and elevated blood pressure (10). Pathological stiffening and structural remodeling of the arterial intima-media layer may occur as early as during childhood (11). Ultrasound-based parameters of carotid stiffness (cS) and intima-media thickness (cIMT) are currently the most extensively validated non-invasive biomarkers to visualize these alterations in minors and young adults (12). As far as we know, only studies with confined samples exist, and there is a lack of evidence about the utility of cIMT and cS in epidemiological settings for population based atherosclerotic risk assessment.

Physical activity, and particularly exercise in terms of recreational or organized sports participation, slow down the progression of vascular aging independent from improvements of the before mentioned traditional risk factors (13, 14). Previous studies indicate, that lack of exercise or even inactivity in childhood may translate into increased progression of cIMT and cS in adulthood (15). Regular exercise and vigorous physical activity on the other hand, are favorably associated with lower cS in children (16) and with thinner aortic IMT in a confined sample of adolescents (17) with elevated cardiovascular risk. In healthy individuals, already modest increases of exercise during childhood may sustainably slow down the progression of arterial stiffening and thickening of the arterial intima-media layer in early (18) and even later adulthood (19). However, the latter studies did not adjust for blood pressure, which is a major determinant of cIMT. Whereas, cross-sectional exercise studies frequently reported no effects of exercise on cS and cIMT (20, 21), longitudinal studies found associations of acute exercise at young age with cS and cIMT in middle aged and older adults (22). The few studies that examined the effects of changes in regular exercise during adolescence on carotid wall function and structure in young adulthood deliver inconsistent evidence about short-term effects of exercise interventions on carotid-arterial stiffening and remodeling in the healthy general population with low cardiovascular risk (18, 23). In summary, existing evidence about effects of exercise on progression of cIMT and cS in adolescents and young adults is limited and it remains unclear, whether exercise during adolescence, in terms of recreational or organized sports participation, leads to measurable adaptations in cS and cIMT in early adulthood. Most importantly, however, the associations of exercise with cIMT and cS have not been examined in a representative population based sample. Therefore, this study analyzed these associations in such a sample and assessed the associations of regular exercise and its changes during adolescence on cIMT and cS in young adulthood.

## Materials and Methods

This prospective study was approved by the German Federal Commissioner for Data Protection and Freedom of Information. Approval was given by the Ethics committees of Medizinische Hochschule Hannover (No. 2275-2014). Informed consent was obtained from all participants in advance.

### Study Sample

**Figure 1:**
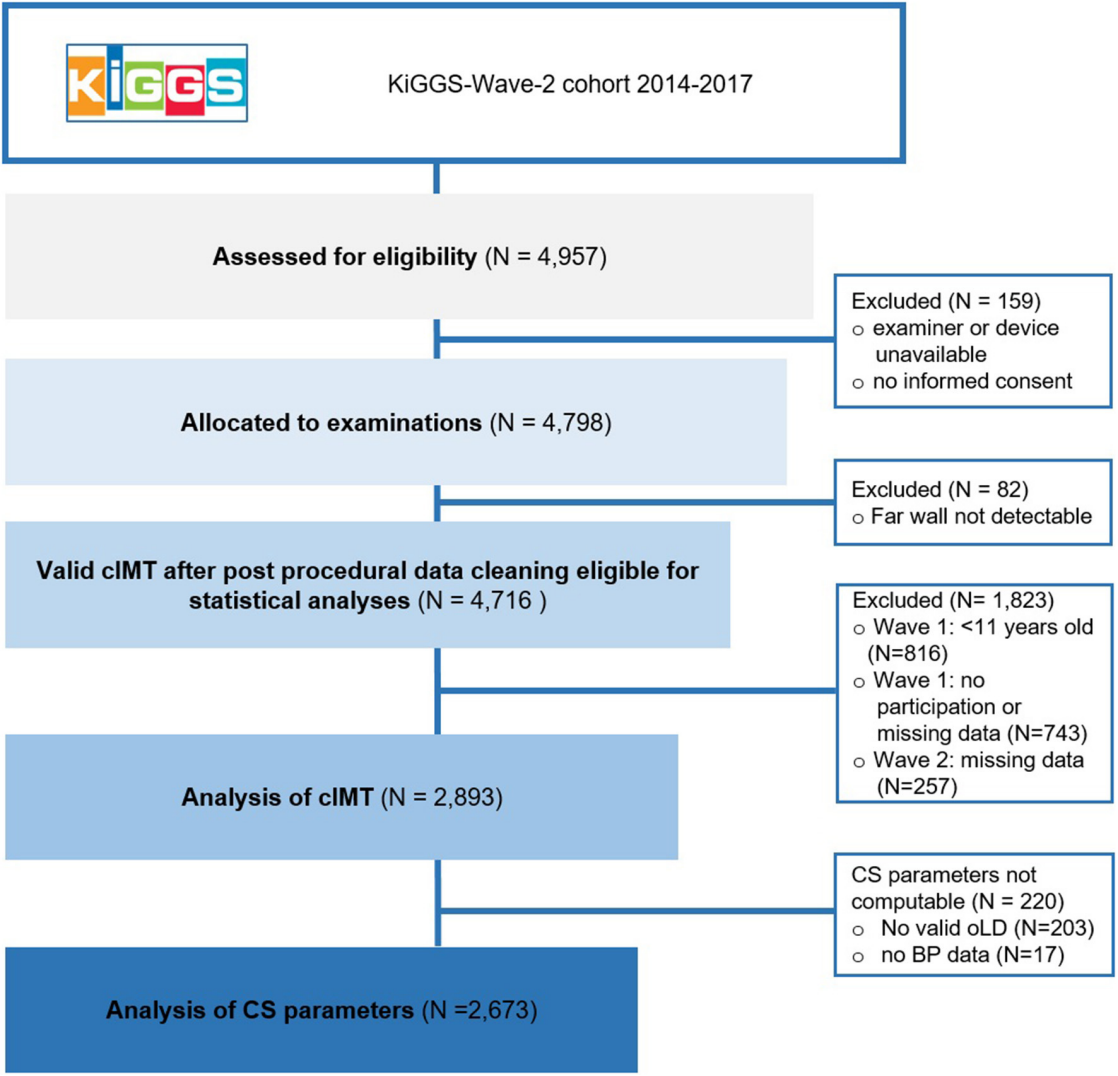
Flow-chart of data collection and cleaning (*n* unweighted).

The study sample comprised 14–28 year old participants of the second follow-up survey KiGGS-Wave-2 of the “German Health Interview and Examination Survey for Children and Adolescents” (24, 25) (Figure 1). Being the first nationwide representative health survey among German children and adolescents, KiGGS was initially conducted in 167 communities between 2003 and 2006. The first follow-up, KiGGS-Wave-1, was conducted between 2009 and 2012 and the second, KiGGS-Wave-2, was conducted from 2014 until 2017. Questionnaire-based assessment of exercise habits was performed at KiGGS-Wave-1 and KiGGS-Wave-2. Measurement of cIMT and cS in B-mode ultrasound sequences was conducted at KiGGS-Wave-2 in 4,798 participants.

### Measurement of Carotid Intima-Media Thickness and Stiffness

Seven centrally trained, certified, and repeatedly retrained physicians conducted the ultrasound examinations. Ultrasound examinations and data acquisition were performed semi-automatically using a portable state-of-the-art ultrasound system (UF-760AG, Fukuda Denshi Co. Ltd., Tokyo, Japan). Standard operating procedures, including computation of cIMT and cS parameters, adhered to current guidelines (26, 27) and have been previously described in detail (28). Far wall common carotid IMT and outer lumen diameter were measured bilaterally in two planes (ear-to-ear and horizontal) within a 10 mm segment 10–15 mm proximal to the carotid bifurcation. The measurement was accepted, if cIMT was validly detected in at least one plane over two consecutive heart cycles. All valid values were averaged to a participant-specific cIMT. Computation of cS parameters was possible when blood pressure as well as valid outer lumen diameter at peak systole and end diastole were available (Figure 1). The equations used for calculation of distensibility coefficient, Young's elastic modulus, Peterson's elastic modulus and β-stiffness index as well as rates of completeness (89%) are presented in detail elsewhere (28).

### General Cardiovascular Risk and Anthropometric Measures

All participants had cardiovascular and metabolic phenotyping based on interviews and medical examinations (25) including metabolic characteristics and risk behavior. Cardiovascular and metabolic profiling included body mass index (by percentile, according to the International Obesity Task Force) (29), resting heart rate (30) and blood pressure (DatascopeAccutorr Plus, Mahwah, NJ) (31), as well as blood analyses of total-, HDL-, and LDL-cholesterol, HbA1c and high-sensitive C-reactive protein. Risk behavior included hazardous drinking, according to the Alcohol Use Disorder Identification Test (32) and current smoking (33). An index of cardiovascular risk (CV-R) was calculated that summarizes z-values based on the KiGGS-Wave-2 study cohort of mean arterial pressure, triglycerides, total/HDL-cholesterol-ratio, body mass index and HbA1c (34).

### Physical Activity Questionnaires

Weekly hours of exercise were assessed at KiGGS-Wave-1 *via* a telephone-based interview and at KiGGS-Wave-2 *via* a self-reporting questionnaire based on the MoMo physical activity questionnaire (35). Participants reported regular weekly hours of exercise referring to recreational and organized sports other than school sports. Categories were “none,” “ <2 h,” “2–4 h,” or “≥4 h” of exercise per week. Based on these categories, cumulative exercise at both measurement points (KiGGS-Wave-1 and KiGGS-Wave-2) as an estimate for exercise trajectories during adolescence and young adulthood were expressed as “low-low exercise” (always <2 h), “low-high exercise” (<2 h at KiGGS-Wave-1 and ≥2 h at KiGGS-Wave-2), “high-low exercise” (≥2 h at KiGGS-Wave-1 and <2 h at KiGGS-Wave-2), “high-high exercise” (always ≥2 h).

### Statistical Analyses

Descriptive characteristics of the sample stratified by weekly hours of exercise at follow-up were presented as either means (± standard deviation) or proportions. To take drop-out and selective re-participation as well as population characteristics into account, weighting factors were used as described elsewhere in detail (25). Both, elevated cIMT and cS, were defined as values ≥90th centile. These are higher than the centiles issued by other recent studies on risk factors of subclinical atherosclerosis in adolescence (12, 36), which have more selective populations. Linear and log-binomial regressions were performed with Stata SE 14.2 (Stata Corp., College Station, TX, US, 2015). Log-binomial regression was performed instead of logistic regression as the odds ratios obtained by the latter overestimate the relative risk when outcomes are common (37). Because elevated cIMT and arterial stiffness as outcomes are derived from sex- and age-specific centiles, the relative risks are all adjusted for sex and age. In addition, log-binomial and logistic regressions were adjusted for height and mean arterial pressure. This decision was based on evidence suggesting that age, sex and height are major determinants of cIMT and cS (38), whereas systolic and diastolic blood pressure seem to be strong confounders of associations involving these vascular biomarkers (39). 95%–confidence intervals are reported to assess the precision of our estimates.

## Results

### General Population Characteristics

General population characteristics are presented in Table 1. Participants' age ranged from 11 to 24 years at KiGGS-Wave-1 and 14–28 years at KiGGS-Wave-2 and was equally distributed. Biomarkers of vascular function and structure as well as general cardiovascular and metabolic factors were not significantly different between groups of exercise, only LDL-cholesterol levels and resting heart rate were lower in the highly active groups (*p* < 0.001). The proportion of active smokers was significantly higher in the “no exercise” group, whereas the proportion of hazardous drinking was highest in the most active group. Mean exercise time decreased from KiGGS-Wave-1 to KiGGS-Wave-2, as indicated by a higher proportion of participants reporting “no exercise” at KiGGS-Wave-2 compared to KiGGS-Wave-1 (26.9 vs. 14.2%), and by a higher proportion of participants reporting less exercise than reporting more exercise at KiGGS-Wave-2 compared to KiGGS-Wave-1 (36.7 vs. 24.9%). The proportion of participants reporting high volumes of exercise (“≥4 h”) was similar in KiGGS-Wave-2 compared to KiGGS-Wave-1 (33.0%). Details about proportions of changes in exercise habits from KiGGS-Wave-1 to KiGGS-Waver-2 are provided in the Supplementary Table 1 in Supplementary Material.

**Table 1:** Characteristics of the study sample.

**Weekly exercise time at KiGGS-wave-2**	**All**	**None**	**<2 h**	**2–4 h**	**≥4 h**
**KiGGS-Wave-1 (** ***N*** **)**	2,893	698	620	573	1,002
Age [years]	17.18 ± 3.75	17.74 ± 3.58	17.29 ± 3.66	17.13 ± 0.55	16.69^***^ ± 0.35
Females (%)	49.64	57.91	60.41	55.14	34.89^***^
**KiGGS-Wave-2 (** ***N*** **)**	2,893	698	620	573	1,002
Age [years]	22.02 ± 3.75	22.54 ± 3.56	22.13 ± 3.69	21.95 ± 3.84	21.55^***^ ± 3.84
cIMT [mm]	0.55 ± 0.05	0.54 ± 0.05	0.54 ± 0.05	0.54 ± 0.05	0.55 ± 0.05
Wall-to-lumen-ratio	0.09 ± 0.01	0.09 ± 0.01	0.09 ± 0.01	0.09 ± 0.01	0.09 ± 0.01
β-SI [–]	4.08 ± 0.86	4.07 ± 0.79	4.03 ± 0.88	4.06 ± 0.89	4.14 ± 0.89
DC [10^−3^ kPa]	45.03 ± 11.35	45.02 ± 10.48	45.78 ± 10.87	45.57 ± 12.17	44.22 ± 11.81
EP [kPa]	50.65 ± 12.03	50.55 ± 11.29	49.60 ± 11.55	50.25 ± 12.97	51.64 ± 12.29
YEM [kPA]	0.28 ± 0.08	0.28 ± 0.08	0.28 ± 0.08	0.28 ± 0.08	0.29 ± 0.09
CV-R [–]	0.40 ± 3.25	0.85 ± 3.93	0.36 ± 2.67	0.17 ± 3.10	0.19 ± 2.90
Body mass index [kg/m^2^]	24.02 ± 4.59	24.33 ± 4.89	23.97 ± 4.44	24.04 ± 5.01	23.78 ± 4.07
MAP [mmHg]	92.07 ± 8.63	92.17 ± 8.83	91.66 ± 8.67	91.57 ± 8.87	92.55 ± 8.19
HbA1c [mmol/mol]	32.66 ± 4.89	32.97 ± 6.85	32.50 ± 2.75	32.30 ± 3.17	32.72 ± 4.52
Triglycerides [mg/dL]	1.30 ± 0.95	1.40 ± 1.19	1.32 ± 0.74	1.30 ± 0.87	1.21^*^ ± 0.85
Total-/HDL-chol. ratio [–]	3.35 ± 0.88	3.49 ± 0.98	3.38 ± 0.84	3.29^*^ ± 0.82	3.25^***^ ± 0.81
LDL-cholesterol	2.60 ± 0.77	2.72 ± 0.80	2.66 ± 0.74	2.56^*^ ± 0.76	2.49^**^ ± 0.73
Resting heart rate [bpm]	75.10 ± 11.65	78.32 ± 10.75	76.81 ± 11.20	73.98^***^ ± 12.06	72.01^***^ ± 11.53
hsCrP [mg/dL]	2.35 ± 4.20	2.44 ± 4.22	2.64 ± 4.39	2.63 ± 5.22	1.93 ± 3.22
Overweight or Obesity^&^ (%)	14.36	17.82	13.12	14.90	12.03^*^
Current smoking (%)	30.94	40.40	24.58^***^	28.05^**^	28.98^**^
Hazardous drinking^#^ (%)	28.01	21.94	26.87	29.20	33.08^***^

*Data presented as weighted mean ± standard deviation (SD), unless specified otherwise. Significance codes*:

* *p < 0.05*,

** *p < 0.01*,

*** *p < 0.001*.

& *Based on KiGGS (40)*.

# *Hazardous drinking (32): AUDIT-C >5 (male)/4 (female)*.

*cIMT, carotid intima-media thickness; β-SI, ß stiffness index; DC, distensibility coefficient; EP, Peterson's elastic modulus; YEM, Young's elastic modulus; CV-R, Index of cardiovascular risk (sum of z-scores of mean arterial pressure, triglycerides, total/HDL-cholesterol-ratio, body mass index and HbA1c). MAP, mean arterial pressure; HbA1c, glycated hemoglobin; hsCrP, high-sensitive C-reactive protein; bpm, beats per minute*.

### Association of Exercise With Cardiovascular Risk

**Figure 2:**
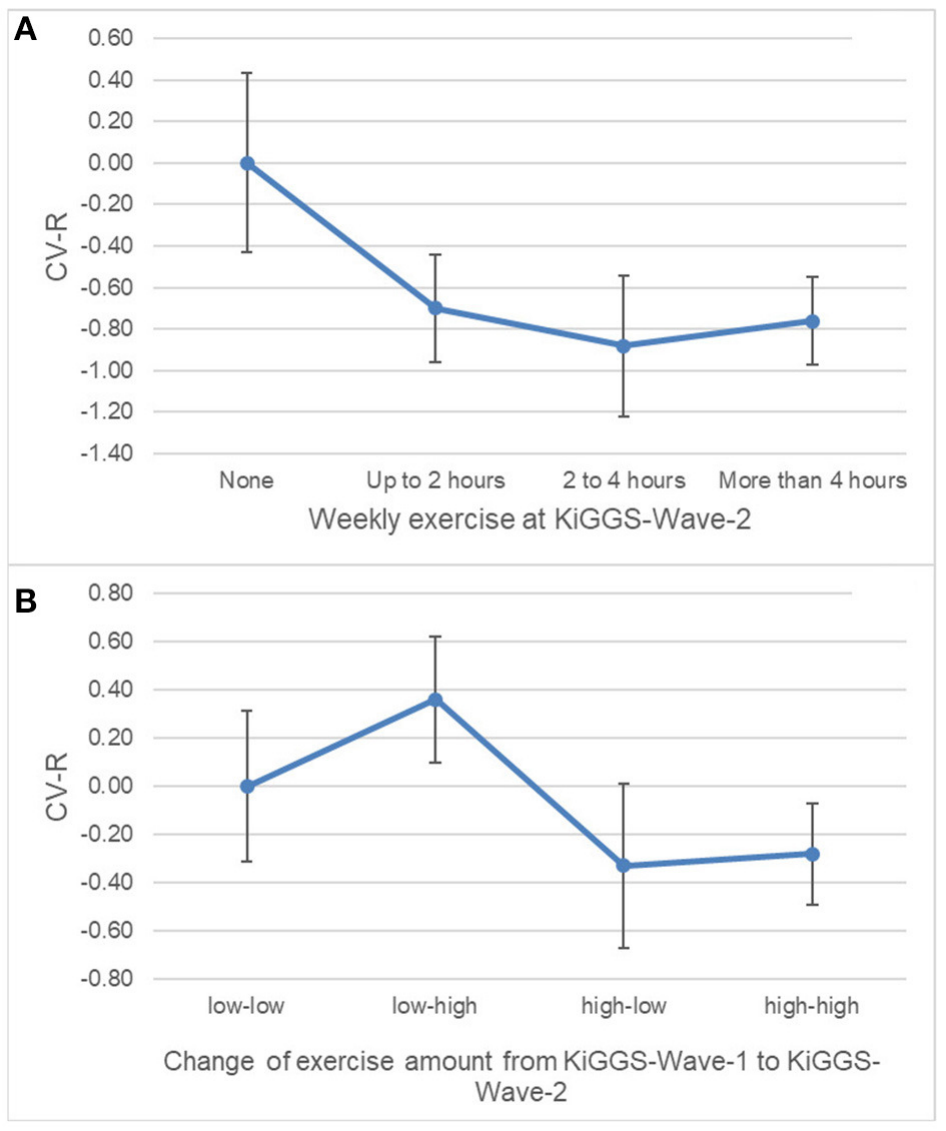
Margin plots demonstrating associations of the cardiovascular risk index (CV-R) with cross-sectional **(A)** and longitudinal **(B)** volumes of exercise. CV-R is significantly lower with higher cross-sectional and longitudinal volumes of exercise. “None” set as reference. Weighted estimations from linear regression; *n* = 2,893. Vertical bars = 95%–confidence intervals. Models adjusted for sex and age.

**Figure 3:**
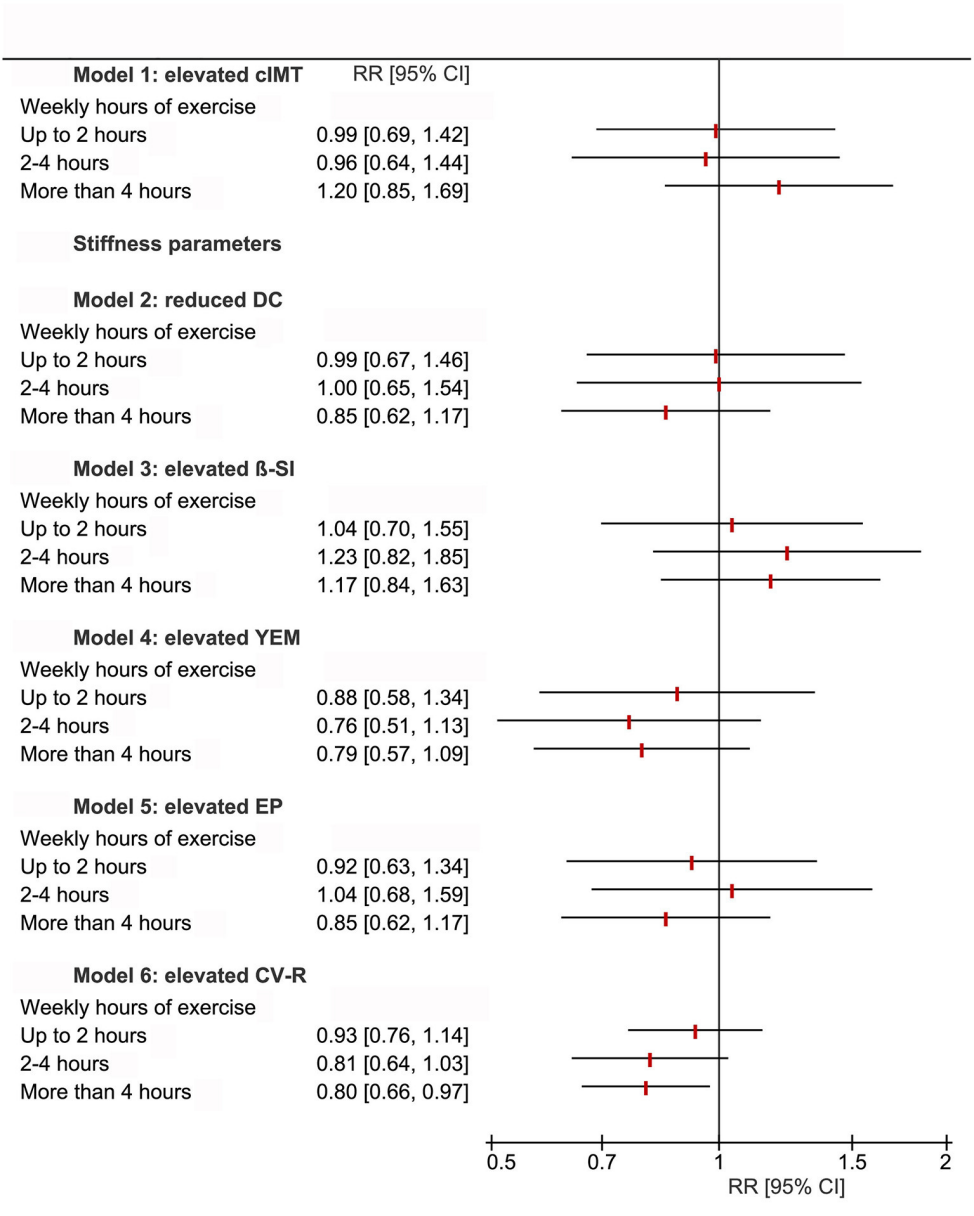
Forest plot demonstrating relative risk for intima-media thickness (cIMT ≥ 90th percentile), parameters of carotid stiffness (cS ≥ 90th percentile) and elevated cardiovascular risk (CV-R ≥ 1 standard deviation) stratified by cross-sectional exercise at KiGGS-Wave-2. Relative risk for elevated CV-R is significantly lower with higher volumes of cross-sectional exercise. No between-group differences were observed for relative risk of elevated cS parameters and cIMT. Results from log binomial regression models. If the 95% confidence interval does not include the null value (RR = 1), the finding is statistically significant. Reference level of exposure: no regular exercise; weighted analyses. DC, distensibility coefficient; β-SI, ß stiffness index; YEM, Young's elastic modulus; EP, Peterson's elastic modulus; CV-R, Index of cardiovascular risk (sum of *z*-scores of mean arterial pressure, triglycerides, total/HDL-cholesterol-ratio, body mass index, and HbA1c); RR [95%-CI], Relative risk [95%–confidence interval].

**Figure 4:**
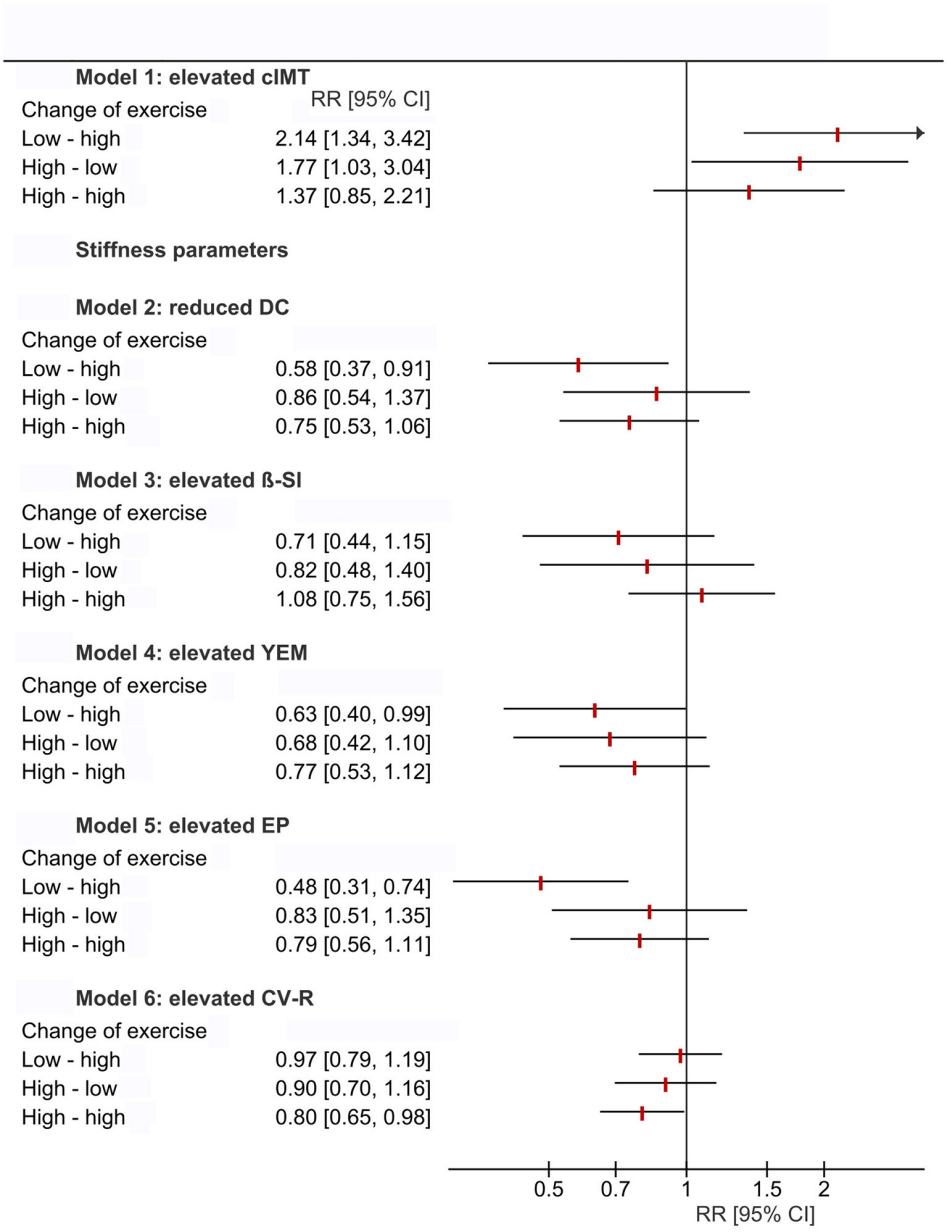
Forest plot demonstrating relative risk for intima-media thickness (cIMT ≥ 90th percentile), parameters of carotid stiffness (cS ≥ 90th percentile) and elevated cardiovascular risk (CV-R ≥ 1 standard deviation) stratified by longitudinal changes of exercise between KiGGS-Wave-1 and KiGGS-Wave-2. Relative risk for elevated CV-R is significantly lower with regularly higher volumes of exercise, as is the relative risk for elevated cS parameters in tendency. No between-group differences were observed for relative risk of elevated cIMT. Results from log binomial regression models. If the 95% confidence interval does not include the null value (RR = 1), the finding is statistically significant. Changes of exercise from KiGGS-Wave-1 to KiGGS-Wave-2: “low-low” (always <2 h), “low-high” (<2 h at KiGGS-Wave-1 and ≥2 h at KiGGS-Wave-2), “high-low” (≥2 h at KiGGS-Wave-1 and <2 h at KiGGS-Wave-2), “high-high” (always ≥2 h); Reference level of exposure: “low-low”; weighted analyses; DC, distensibility coefficient; β-SI, ß stiffness index; YEM, Young's elastic modulus; EP, Peterson's elastic modulus; CV-R, Index of cardiovascular risk (sum of *z*-scores of mean arterial pressure, triglycerides, total/HDL-cholesterol-ratio, body mass index and HbA1c); RR [95%-CI], Relative risk [95%–confidence interval].

CV-R was significantly lower with higher cross-sectional (B = −0.73, 95%-CI = −1.26 to 0.19, *p* = 0.008) and longitudinal (B = −0.37, 95%-CI = −0.74 to 0.00, *p* = 0.048) exercise (Figure 2; Supplementary Tables 2, 3 in Supplementary Material). Relative risk for elevated CV-R was significantly lower in all exercise-groups compared to “no exercise” (RR = 0.80, 95%-CI = 0.66–0.98, *p* = 0.033) (Figure 3). High volumes of exercise at both measurement points were associated with lower relative risk of elevated CV-R (RR = 0.80, 95%-CI = 0.65–0.97, *p* = 0.021) (Figure 4).

### Association of Exercise With Vascular Structure and Function

There were no between-group differences of cIMT and cS for cross-sectional and also longitudinal exercise (Supplementary Tables 2, 3 in Supplementary Material). There were also no between-group differences of relative risk for elevated cIMT and cS for cross-sectional exercise. Relative risk for elevated cS was lower in tendency with high volumes of exercise at both measurement points, whereas relative risk for elevated cIMT showed no between-group differences (Figures 3, 4).

## Discussion

In summary, the study results of the analysis of a representative, so far largest cohort of adolescents and young adults demonstrate a more favorable constellation of classical atherosclerotic risk factors as well as a lower risk for an early increase of cS in healthy individuals who engage in regular exercise. However, biomarkers of vascular function and structure measured with state-of-the-art ultrasound equipment and vascular analysis software were not significantly associated with regular short- (cross-sectional) and long-term (longitudinal) exercise, contradicting previous findings (18). This might raise doubts for the utility of cS and cIMT to monitor effects of exercise on vascular atherosclerotic changes at such a young age.

On the other hand, our results confirm existing evidence (41, 42) demonstrating that physical activity, particularly exercise at higher intensities, may favorably influence atherosclerotic risk. However, the extent to which exercise can decelerate the progression of atherosclerotic changes may depend on the chronic cumulative exposure toward exercise rather than on high volumes in the short-term (43). Whereas, short-term exercise interventions lasting several months found only little or no effects of resistance training on carotid wall structure in young (21) and of aerobic training in older subjects (20), long-term interventions lasting at least 4 years demonstrated a slower progression of functional and structural vascular decline in regularly active, older individuals (44, 45). Yet again, only one small short-time exercise study was found that assessed the effects of 1 year recreational or organized sports participation on cIMT, but not cS, in adolescents (46). Accordingly, the current study adds valuable evidence.

The assessment of vascular protective effects of exercise on the in the general healthy population may have to take the chronological course of vascular aging into account. Results from our study agree with those from a smaller cohort in the European Youth Heart Study indicating, that parameters of vascular function and structure in children might remain independent from a child's activity level, whereas atherosclerotic risk factors may be favorably altered in those being exposed to regular exercise (47). That does not implicate the ineffectiveness of regular exercise in terms of protecting vascular health, but rather might reflect the compensatory capacities of a juvenile organism toward an unhealthy lifestyle. Results from the Amsterdam Growth and Health Longitudinal Study (22) and the Cardiovascular Risk in Young Fins Study (19) indicate that in middle-aged adults, functional biomarkers, such as cS, are significantly different in those with a lifelong high exercise-level compared to low-active peers. Thus, cIMT and cS might be indicators of a slower progression of vascular aging due to long-term exposure to regular exercise in adults rather than an accelerated progression of vascular aging in sedentary adolescents.

The lack of association between cIMT and exercise in our study stands in contrast to another study (17) that found a slower progression of aortic IMT with higher volumes of intensive physical activity already during adolescence. However, peripheral arteries, such as the carotid artery, are inherently stiffer than the aortic artery and their age-related stiffening may be less marked (48). Thus, measurement of central arterial stiffness and wall thickness might be more indicative for exercise-related vascular effects than cS and cIMT in adolescents and young adults.

### Practical Considerations

Our study results on a population-based cohort confirm data from previous studies on selected samples, that a high volumes of regular exercise (>4 h/week) during adolescence and young adulthood, play a vital part in the maintenance of low atherosclerotic risk in the healthy general population. This effect most likely translates into lower cardiovascular morbidity and mortality later in life (2). In addition, the comparison of interventional studies in young (49, 50) vs. older (20, 51) individuals suggests higher cardiovascular adaptive responsiveness toward exercise programs in the young when as much as 95% of cardiovascular risk appear modifiable (17, 52). Furthermore, patterns of physical activity during childhood and adolescence track into adulthood remaining more or less stable along the life course (53). Pediatricians, but also other clinicians and health care providers, should thus be encouraged to promote the participation in regular recreational and organized sports of ≥4 h per week at the youngest possible age.

### Methodological Considerations

A clear strength of this study is the assessment of multiple biomarkers of carotid wall structure and function. However, we only measured cIMT and cS of the common but not of the internal carotid artery or the carotid bulb, because of its higher visibility, completeness, procedural standardization and predictive value (54–56). Screening of the carotid tree for atherosclerotic plaques was not conducted because of an extremely low prevalence in adolescents and young adults (57). Furthermore, it has to be kept in mind, that exercise-related vascular adaptations may show some degree of local variation, due to distinct shear patterns induced by the type of exercise and the area of exposure (58). Especially in individuals engaged in sports predominantly involving the lower extremities favorable local vascular effects might not be detected by carotid arterial ultrasound. The application of a third-generation cIMT detection software, which includes automatic quality control already during image acquisition, promotes the thorough adjustment of the measurement plane by the sonographer. Together with the extensive post-procedural quality control and the rigorous adherence to current guidelines (12), this implies the currently highest quality of cIMT and cS data in a large representative population sample. We did not adjust for the ethnic background of study participants, as the sample was very homogeneous, with more than 95% Caucasians and because the relevance of ethnicity and geographical factors, such as latitude, for cIMT-related atherosclerotic risk assessment is likely to be rather low (59).

The assessment of physical activity based on self-reported questionnaires is a limitation. For validation purposes, volume of exercise in terms of moderate-to-vigorous physical activity was assessed via accelerometer in a subsample of our study cohort at KiGGS-Wave-2, showing significant correlations with questionnaire-based exercise. Furthermore, due to feasibility reasons, exercise habits could be assessed only twice during the study period. However, as exercise habits that have been developed during childhood tend to remain stable until adulthood (53), we assume that this sufficiently characterizes participants according to their long-term exercise habits. This is indirectly supported by lower levels of LDL-cholesterol and resting heart rate in the highly active groups with 2–4 and more than 4 h of weekly exercise. In addition, assessment of physical activity in this study was based on the MoMo physical activity questionnaire (35), which show good validity and reliability specifically for physical activity at higher intensities, such as sport exercise.

We did not adjust our models for maturation status, as there is currently no evidence supporting a relevant effect on cIMT and cS in models accounting for the strongest confounders (age, sex, height, carotid lumen diameter, and blood pressure) (60, 61).

### Conclusions and Perspectives

Increasing and maintaining high levels of physical activity are associated with a better cardiovascular risk profile in adolescents and young adults. However, data available so far including this first study based on a representative national cohort show no association with common carotid structural and functional biomarkers. This study demonstrates, that on a population level exercise may contribute to an overall favorable constellation of atherosclerotic risk factors already during adolescence and young adulthood, if applied regularly over several years. The measurement of cS and cIMT for monitoring needs and success of preventive measures at such a young age and in a population sample with low atherosclerotic risk may be questioned. Despite great improvements in measuring techniques for these vascular biomarkers, exercise-related changes in the young and healthy individuals are subtle. Studies with longer follow-up periods, repeated measurements and more granular risk factor trajectories including lifestyle behavior are needed to further investigate the variable utility of vascular biomarkers throughout the life course. Yet, from a practical point of view, adolescents and young adults should be encouraged to engage in regular exercise according to the WHO guidelines on physical activity and sedentary behavior of at least 2 h or more per week and avoid periods of inactivity in order to maintain optimal cardiovascular health (62).

## Data Availability Statement

The datasets presented in this article are not readily available because of restrictions due to data protection policies. Requests to access the datasets should be directed to NeuhauserH@rki.de.

## Ethics Statement

The studies involving human participants were reviewed and approved by Medizinische Hochschule Hannover (No. 2275-2014). Written informed consent to participate in this study was provided by the participants' legal guardian/next of kin.

## Author Contributions

KK wrote the manuscript, conducted data analysis, and participated in the conception and conduction of the study. JB participated in the conception of the study, data analysis, and revision of the manuscript drafts. GS and SK participated in the conduction of the study, extensively engaged in preparation, and revision of the manuscript drafts. HN and AS-T participated in the conception of the study, supervised the conduction and data analysis, extensively engaged in preparation, and revision of the manuscript drafts. All authors contributed to the article and approved the submitted version.

## Funding

The KiGGS study was funded by the German Federal Ministry of Health and the Robert Koch-Institute.

## Conflict of Interest

The authors declare that the research was conducted in the absence of any commercial or financial relationships that could be construed as a potential conflict of interest.

## Publisher's Note

All claims expressed in this article are solely those of the authors and do not necessarily represent those of their affiliated organizations, or those of the publisher, the editors and the reviewers. Any product that may be evaluated in this article, or claim that may be made by its manufacturer, is not guaranteed or endorsed by the publisher.

## Abbreviations

cIMT: carotid intima-media thickness
cS: carotid stiffness
CV-R: Index of cardiovascular risk
exercise: recreational and organized sports participation.
