# Supplementary material for: Exercise and Carotid Properties in the Young–The KiGGS-2 Study
Source: Front Cardiovasc Med. 2022 Jan 5;8:767025. doi: 10.3389/fcvm.2021.767025 (PMC8766972; doi:10.3389/fcvm.2021.767025)
Supplement: Supplementary file 1 [file Data_Sheet_1.pdf]

Supplementary Material

Table S1: Track of weekly exercise between KiGGS-Wave-1 and KiGGS-Wave-2. At KiGGS-Wave 2, 24.9% of all participants reported higher amounts, 38.5% reported no change and 36.7% reported lower amounts of exercise than at KiGGS-Wave-1.

Weekly exercise at KiGGS-Wave-1	Weekly exercise at KiGGS-Wave-2				
	none	<2 hours	2 - 4 hours	≥4 hours	Total
none	7.15%	3.12%	1.73%	2.19%	14.18%
<2 hours	10.30%	7.04%	5.02%	5.21%	27.57%
2 - 4 hours	5.11%	6.01%	6.20%	7.59%	24.90%
≥4 hours	4.29%	5.14%	5.85%	18.06%	33.34%
Total	26.85%	21.31%	18.80%	33.04%	100%

Notes: n = 2,893; weighted estimations; Blue color = higher amounts of exercise at KiGGS-Wave-2; Yellow color = no change of exercise at KiGGS-Wave-2; Orange color = lower amounts of exercise at KiGGS-Wave-2.

Table S2: Weighted multivariable linear regressions of associations of carotid intima-media thickness (cIMT), parameters of carotid stiffness (cS) and cardiovascular risk (CV-R) with cross-sectional exercise at KiGGS-Wave-2. CV-R was lower with high amounts of cross-sectional exercise (4+ hours) than with no regular exercise. No such associations were observed for cIMT and parameters of cS.

	Exercise at KiGGS-Wave-2		
	up to 2 hours	2-4 hours	4+ hours
**cIMT**			
Coefficient estimate	-0.01	0.03	0.04
95% Confidence Interval	[-0.16, 0.14]	[-0.12, 0.17]	[-0.10, 0.18]
**cS**			
DC			
Coefficient estimate	0.02	0.00	-0.03
95% Confidence Interval	[-0.13, 0.16]	[-0.15, 0.15]	[-0.16, 0.09]
β-SI			
Coefficient estimate	-0.01	0.01	0.05
95% Confidence Interval	[-0.16, 0.15]	[-0.14, 0.17]	[-0.08, 0.18]
YEM			
Coefficient estimate	-0.01	-0.01	0.00
95% Confidence Interval	[-0.17, 0.14]	[-0.15, 0.13]	[-0.13, 0.13]
EP			
Coefficient estimate	-0.02	0.00	0.03
95% Confidence Interval	[-0.17, 0.13]	[-0.15, 0.14]	[-0.09, 0.16]
**CV-R**			
Coefficient estimate	-0.40	-0.57	-0.73**
95% Confidence Interval	[-0.93, 0.13]	[-1.14, 0.00]	[-1.26, -0.19]

Notes: Reference category: no regular exercise at KiGGS-Wave-2. Adjusted for sex, age, height and mean arterial pressure. DC= distensibility coefficient. β-SI= ß-stiffness index. YEM= Young's elastic modulus. EP= Peterson's elastic modulus. Significance codes: * = $p<0.05$, ** = $p<0.01$, *** = $p<0.001$.

Table S3: Weighted multivariable linear regressions of associations of carotid intima-media thickness (cIMT), parameters of carotid stiffness (cS) and cardiovascular risk (CV-R) with trajectories of exercise from KiGGS-Wave-1 to KiGGS-Wave-2. Only with regularly high amounts of exercise ("high-high") CV-R was significantly lower than with regularly low amounts of exercise ("low-low"). No such associations were found for parameters of cS and cIMT. The tendency towards a higher cIMT with trajectories of higher amounts of exercise ("low-high" and "high-high") displays a small effect. However, this might indicate a tendency towards an adaptive increase of cIMT in individuals who have been physically active for many years.

	**Exercise trends**		
	Low - high	High - low	High - high
**cIMT**			
Coefficient estimate	0.19*	0.11	0.13*
95% Confidence Interval	[0.04, 0.34]	[-0.06, 0.28]	[0.00, 0.26]
**cS**			
DC			
Coefficient estimate	0.09	0.00	0.01
95% Confidence Interval	[-0.06, 0.23]	[-0.15, 0.15]	[-0.12, 0.14]
β-SI			
Coefficient estimate	-0.10	-0.04	0.01
95% Confidence Interval	[-0.26, 0.07]	[-0.19, 0.11]	[-0.13, 0.15]
YEM			
Coefficient estimate	-0.16*	-0.08	-0.06
95% Confidence Interval	[-0.31, -0.01]	[-0.23, 0.08]	[-0.20, 0.07]
EP			
Coefficient estimate	-0.10	-0.01	-0.02
95% Confidence Interval	[-0.24, 0.05]	[-0.16, 0.14]	[-0.15, 0.12]
**CV-R**			
Coefficient estimate	0.25	-0.43	-0.37*
95% Confidence Interval	[-0.38, 0.88]	[-0.91, 0.06]	[-0.74, 0.00]

Notes: Trajectories of exercise from KiGGS-Wave-1 to KiGGS-Wave-2: "low-low" (always <2 hours), "low-high" (<2 hours at KiGGS-Wave-1 and ≥2 hours at KiGGS-Wave-2), "high-low" (≥2 hours at KiGGS-Wave-1 and <2 hours at KiGGS-Wave-2), "high-high" (always ≥2 hours). Reference level of exposure: "low-low". Adjusted for sex, age, height and mean arterial pressure. β-SI= ß-stiffness index. YEM= Young's elastic modulus. EP= Peterson's elastic modulus. Significance codes: * = p<0.05, ** = p<0.01, *** = p<0.001.
